# Identification and genetic characterization of a minor norovirus genotype, GIX.1[GII.P15], from China

**DOI:** 10.1186/s12863-022-01066-6

**Published:** 2022-07-06

**Authors:** Yanli Chen, Qiongwen Wu, Guiman Li, Hongzhe Li, Wenlong Li, Heng Li, Li Qin, Huiwen Zheng, Changkun Liu, Min Hou, Longding Liu

**Affiliations:** 1 Yunnan Key Laboratory of Vaccine Research and Development on Severe Infectious Diseases, Institute of Medical Biology, Chinese Academy of Medical Science and Peking Union Medical College, No. 935 alternating current Road, Wuhua District, Kunming, 650118 Yunna China; 2 Key Laboratory of Systemic Innovative Research on Virus Vaccine, Chinese Academy of Medical Sciences, Kunming, China; 3 Kunming City Center for Disease Control and Prevention, Kunming, China

**Keywords:** Norovirus, Full-length genome, GIX.1[GII.P15], Phylogenetic analysis

## Abstract

**Background:**

Human noroviruses, single-stranded RNA viruses in the family *Caliciviridae*, are a leading cause of nonbacterial acute gastroenteritis in people of all ages worldwide. Despite three decades of genomic sequencing and epidemiological norovirus studies, full-length genome analyses of the non-epidemic or minor norovirus genotypes are rare and genomic regions other than ORF2 and 3′-end of ORF1 have been largely understudied, which hampers a better understanding of the evolutionary mechanisms of emergence of new strains. In this study, we detected a rare norovirus genotype, GIX.1[GII.P15], in a vomit sample of a 60 year old woman with acute gastroenteritis using Raji cells and sequenced the complete genome.

**Results:**

Using electron microscopy, a morphology of spherical and lace-like appearance of norovirus virus particles with a diameter of approximately 30 nm were observed. Phylogenetic analysis of VP1 and the RdRp region indicated that the KMN1 strain could be genotyped as GIX.1[GII.P15]. In addition, the VP1 region of KMN1 strain had 94.15% ± 3.54% percent nucleotide identity (PNI) compared to 26 genomic sequences available in GenBank, indicating a higher degree similarity between KMN1 and other GIX.1[GII.P15] strains. Further analysis of the full genome sequence of KMN1 strain showed that a total of 96 nucleotide substitutions (63 in ORF1, 25 in ORF2, and 8 in ORF3) were found across the genome compared with the consensus sequence of GIX.1[GII.P15] genome, and 6 substitutions caused amino acid changes (4 in ORF1, 1 in ORF2, and 1 in ORF3). However, only one nucleotide substitution results in the amino acid change (P302S) in the VP1 protein and the site was located near one of the predicted conformational B epitopes on the dimer structure.

**Conclusions:**

The genomic information of the new GIX.1[GII.P15] strain KMN1, which was identified in Kunming, China could provide helpful insights for the study of the genetic evolution of the virus.

**Supplementary Information:**

The online version contains supplementary material available at 10.1186/s12863-022-01066-6.

## Introduction

Human norovirus (HuNoV) is a member of the genus *Norovirus* in the family *Caliciviridae,* and it is one of the most common enteric pathogens causing epidemic gastroenteritis in humans of all ages [1, 2]. The genome sequence of norovirus is ~ 7.6 kb in length, and comprises of three open reading frames (ORFs). ORF1 encodes six nonstructural (NS) proteins including p48, NTPase, P22, VPg, Pro, and Pol which play a critical role in virus replication [3]; ORF2 encodes the major capsid protein (VP1) which consists of a protruding (P) domain and a shell (S) domain [4, 5]. The P domain can be divided into P1 and P2 subdomains, and P2 is the most important factor in determining the diversity, antigenicity, and glycan binding patterns of different types of norovirus. ORF3 encodes the minor capsid protein (VP2) which responsible for capsid assembly and genome encapsidation [6]. Based on the genetic differences within VP1 and RdRp regions, norovirus has been classified into 10 genogroups and more than 40 different genotypes [7].

Of the more than 30 known genotypes to infect humans, GII.4 viruses have been the most prevalent viruses associated with epidemic and endemic norovirus gastroenteritis in the world for over two decades [8, 9]. However, other genotypes such as GII.17, GII.2, GI.3, GII.3, GII.6, GII.12 [10–13], are also frequently reported to cause norovirus illness. GIX.1[GII.P15] viruses have been reported in several countries in recent years including in Saudi Arabia, China and the US [14–16]. Importantly, this genotype was detected as early as 1990 associated with a large outbreak of acute gastroenteritis in US troops deployed to Saudi Arabia [17]. Since then this genotype (until recently known as GII.15) has been detected very rarely. Hence, a larger number of complete genomic GIX.1[GII.P15] sequences are needed to further study the evolution and epidemiology of this genotype.

Here, we report the complete genome of a norovirus GIX.1[GII.P15] strain collected from a gastroenteritis surveillance program performed by the Kunming City Center for Disease Control and Prevention. To better understand the genetic characteristics of this virus, we first isolated this strain in a human B cell culture system and then carried out a comprehensive analysis of the full genome sequence.

## Materials and methods

### Sample information and collection

In this study, vomit sample from a 60-year-old female patient with diarrheal was collected by the Kunming City Center for Disease Control and Prevention in a norovirus surveillance program during winter in Kunming of China in 2017. Informed consent for this study was obtained from the patient, and the protocol was approved by the Ethics Committee of the Institute of Medical Biology, Chinese Academy of Medical Sciences, in accordance with the Declaration of Helsinki. Following the dissolution sample in 2 ml phosphate-buffered saline solution with antibiotics, the vomit supernatant was collected after centrifugation at 12000 × g for 10 min at 4 °C and then kept at − 80 °C.

### Nucleics acids extraction and primary identification

TRIzol Universal Reagent (TianGen Biotech Co., Ltd., Beijing, China) was used to extract the total RNA from 100 μl of vomit supernatant, according to the instructions of the manufacturer. The norovirus genogroup I and II amplification kits (MABSKY, China) was used to primarily identify the genogroup of this strain. For further quantification, real-time TaqMan RT-PCR assay was performed using the one-step PrimeScript™ RT-PCR kit (Takara, Code No. RR064A) in the CFX96 Touch™ Real-Time PCR Detection system (Bio-Rad, Laboratories, Hercules, CA, USA). The PCR reactions were performed by using the forward oligonucleotide primer, 5′-CARGARBCNATGTTYAGRTGGATGAG-3′; reverse primer 5′-TCGACGCCATCTTCATTCACA-3′ and the probe 5’FAM-TGGGAGGGCGATCGCAATCT-TAMRA-3′ [18]. The PCR reaction conditions were as follows: 42 °C for 5 min and 95 °C for 10 s, followed by 40 cycles at 95 °C for 5 s, and 60 °C for 20 s. To generate a standard curve for cycle thresholds (Cts) versus virus copy number, the RNA standards of norovirus containing ORF1 and ORF2 junction region were made by cloning a 813-bp region into the pET-28a vector followed by transcribed in vitro using the Transcript Aid T7 High Yield Transcription Kit (Thermo Scientific, USA). After purification, the RNA transcripts were serially diluted to a range of 10^1^ to 10^12^ copies/μl to build a standard curve. Viral copy number for the sample was calculated based on the standard curve and Ct values of the samples.

### Cell culture, virus isolation and transmission electron microscope observation of viral particles

Raji cells (Human B-lymphocyte cells) were stored in the laboratory and cultured in RPMI-1640 culture medium (Opti-MEM, Thermo Fisher, USA) supplemented with 10% fetal bovine serum (Worthington Biochemical Corporation, USA) and 1% penicillin/streptomycin (pen/strep) in an incubator containing 5% CO_2_ at 37 °C. To isolate the norovirus strain, the vomit supernatant was inoculated in Raji cells in accordance with previously described methods [19]. Briefly, 10^6^ viral genome copies of the vomit sample was added into 2 × 10^5^ Raji cells; then complete RPM1640 was added to top up the mixture to 100 μl and the mixture was incubated for 2 h at 5% CO_2_ at 37 °C; The samples were then centrifuged and the pellet was washed and resuspended using 100 μl of complete RPMI; 50 μl of each sample was added to the 48-well plate, and complete RPMI1640 was added to top up each well to 1 ml, then the plate was incubated in a 37 °C incubator with 5% CO_2_. Virus was collected at 0 and 72 hours and 500 μl aliquots were transferred into two microcentrifuge tubes. To calculate the number of genome copies attached to the B cells, 1 ml TRIzol was added to one aliquot for RNA extraction and the other aliquot was stored for future use.

To facilitate the detection of viral particles, a transmission electron microscope was employed to identify the size and morphology of the norovirus particles. After purification by iodixanol super-centrifugation, the sampled layers containing nanoparticles were applied to formvar-carbon-coated 400-mesh copper grids using a glass microspray device. The grids were stained with 2% aqueous uranylacetate at pH 4.5 for 5 min and viewed under a Hitachi TEM at 10,000–30,000× magnification.

### Full-length genome sequencing and sequence analysis

The full-length genome of the strain cultivated was sequenced by next-generation sequencing (NGS) technology. Briefly, 1 ng of input cDNA was used for library construction with A NEBNext Ultra II RNA Library Prep Kit (NEB, USA). The Illumina MiSeq sequencer (NovaSeq 6000, USA) was used to generate paired-end 150 bp reads. After removing the adapters and trimming from the 3′ end, the sequencing reads were de novo assembled into contigs with SPAdes 3.9.0 [20]. Geneious software package was used to align the nucleotide sequence of KMN1 with other reference strains downloaded from NCBI. MEGA X was used to construct the phylogenetic trees, respectively, based on full-length genome sequences, RdRp and VP1 sequences by using the neighbor-joining method with a Kimura two-parameter model. The bootstrap values were calculated with 1000 replicates. Entropy-One Tool (https://www.hiv.lanl.gov/content/sequence/ENTROPY/entropy_one.html) was used to determine the Shannon entropy values at nucleotide and amino acid level. BioEdit was used to calculate the percent nucleotide identity between KMN1 strain and other GIX.1[GII.P15] strains.

### Creation of the capsid protein structure and prediction of conformational epitopes for the B-cell of the VP1 protein

VP1 dimer structural models of each GIX.1[GII.P15] strain were constructed using SWISS-MODEL Server (The norovirus GIX.1[GII.P15] VP1 dimer structural model of KMN1 strain was constructed by SWISS-MODEL Server). The templates for homology modeling were based on the crystal structures of four strains (PDB ID: 1IHM, 4X07, 4OP7, and 4OPS). Protein structure was visualized and analyzed using the online tool provided by the Swiss Model server. Four bioinformatics tools DiscoTope 2.0 [21], BEPro [22], EPCES [23], and EPSVR [24] were used to predict the conformational epitopes on the capsid VP1 protein of GIX.1[GII.P15] strains. The thresholds for the epitopes were 1.3 for BEPro, − 3.7 for DiscoTope, 2.0 and 70 for EPCES and EPSVR. Conformational epitopes were determined by the consensus sites according to all four tools and regions with similar residues across two of the sites in the VP1 dimer structures.

## Results

### Isolation and identification of the HuNoV

**Fig. 1:**
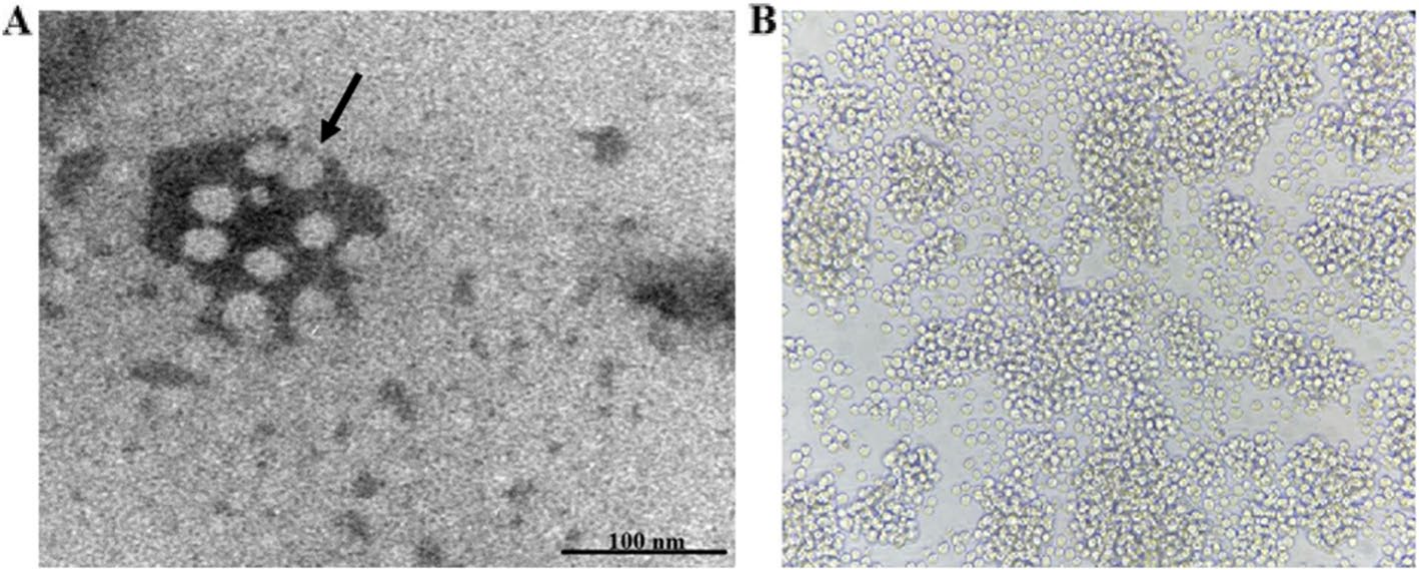
Isolation of KMN1 strain in Raji cells. **A** TEM identifies a 30-nm particle with a morphology of spherical and lace-like appearance associated with KMN1 strain infection (Bar =100 nm). Black arrows indicate aggregates of assembled viral particles. **B** Raji cells were inoculated with 10^6^ genome copies of the indicated HuNoV GIX.1[GII.P15] Vomit samples, and cells were collected at 72 hours after infection

Since previous studies reported that human noroviruses are able to infect and replicate in BJAB and Raji B cell lines [19, 25], we used Raji cells to attempt to isolate norovirus from the vomit sample. The newly isolated virus was named KMN1. To facilitate the detection of viral particles, Raji cells were collected at 48 hours after infection for examination by electron microscope. Electron microscopy identified virus particles with a diameter of approximately 20–40 nm and a morphology of spherical and lace-like appearance within the infected Raji cells, as shown in Fig. 1. Norovirus genome replication was detected using real-time RT-PCR with RNA extracted from the cell culture medium. The input number of virus copies that attached to the B cells was 10^4^ copies/μl, and increased by approximately 4.6-fold to 10^4.66^ copies/μl at 72 hours post-infection (hpi), indicating that primary Raji infection results in the production of new infectious virus particles. Of note, the inoculated cells at 72 hpi were harvested and subjected to next-generation sequencing (NGS) to provide full-length genomic sequences.

### The complete genome and phylogenetic analysis of the KMN1 strain

**Fig. 2:**
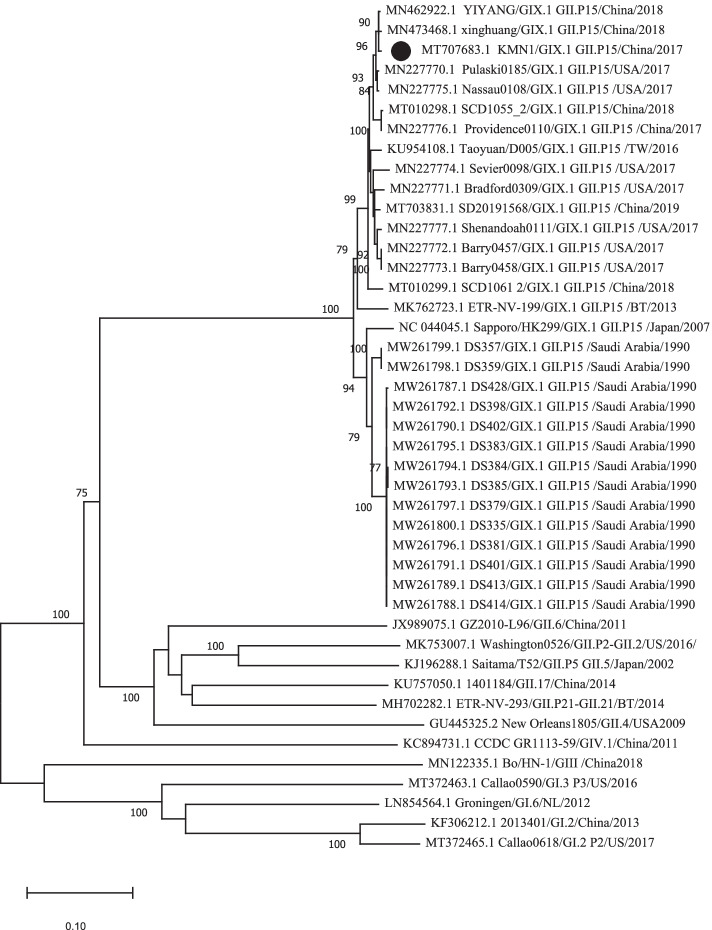
Phylogenetic tree based on full-length VP1 sequences using the neighbor-joining method. The GIX.1[GII.P15] strain identified in this study is indicated with a solid black circle. Bootstrap values greater than 75% are shown on the corresponding branches

**Fig. 3:**
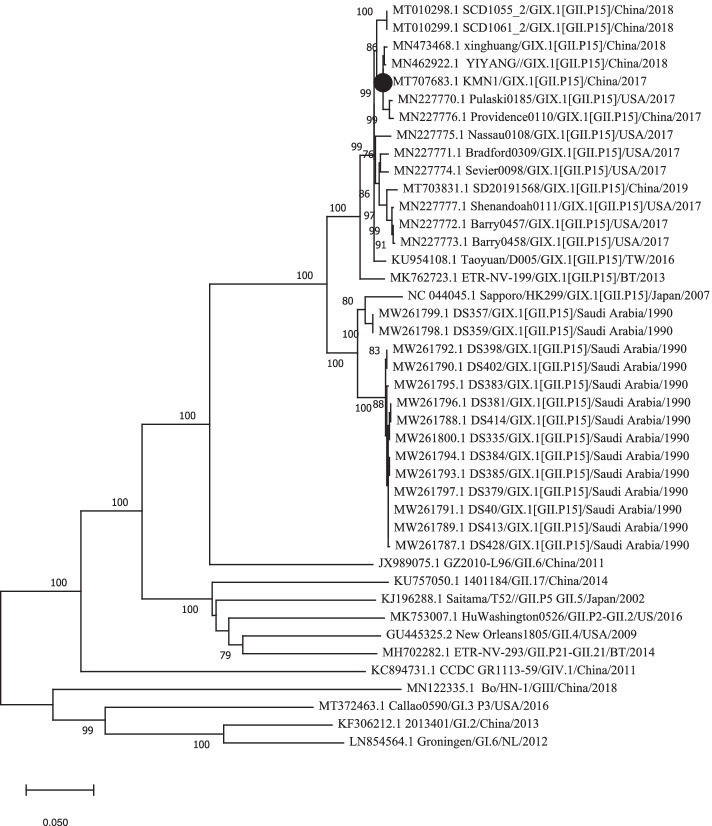
Phylogenetic analysis based on full-length RdRp sequences using the neighbor-joining method. The GIX.1[GII.P15] strain identified in this study is indicated with a solid black circle. Bootstrap values greater than 75% are shown on the corresponding branches

The full genome sequence of KMN1 strain was submitted to GenBank with an accession number of MT707683.1. Similar to other types of norovirus, the genome sequence length of the KMN1 strain was 7594 nt and consisted of three ORFs. To understand the genetic characterization of the KMN1 strain, we first performed the phylogenetic analysis based on the nucleotide sequences of VP1 and RdRp. Results showed that the KMN1 strain have 99.46 and 99.54% identities, respectively, with YIYANG/HUNAN/CHINA/GII.P15-GIX.1/2018 strain (Figs. 2 and 3), both of which were collected in China in early 2018. In addition, phylogenetic trees based on the full-length genome (Supplementary Fig. S1) showed the same results and suggested that no evidence of recombination is found in this novel strain. Within the GIX.1[GII.P15] cluster, norovirus from China and USA from 2017 to 2019 clustered with the KMN1 strain, while the earlier strains collected in 1990 and 2007 formed another subcluster, indicating that the nucleotide sequences of the 2017–2019 GIX.1[GII.P15] strains presented distinct genetic divergence compared with earlier strains collected in 1990 and 2007.

### Alignment analysis of full nucleotide and amino acid sequences of KMN1

**Fig. 4:**
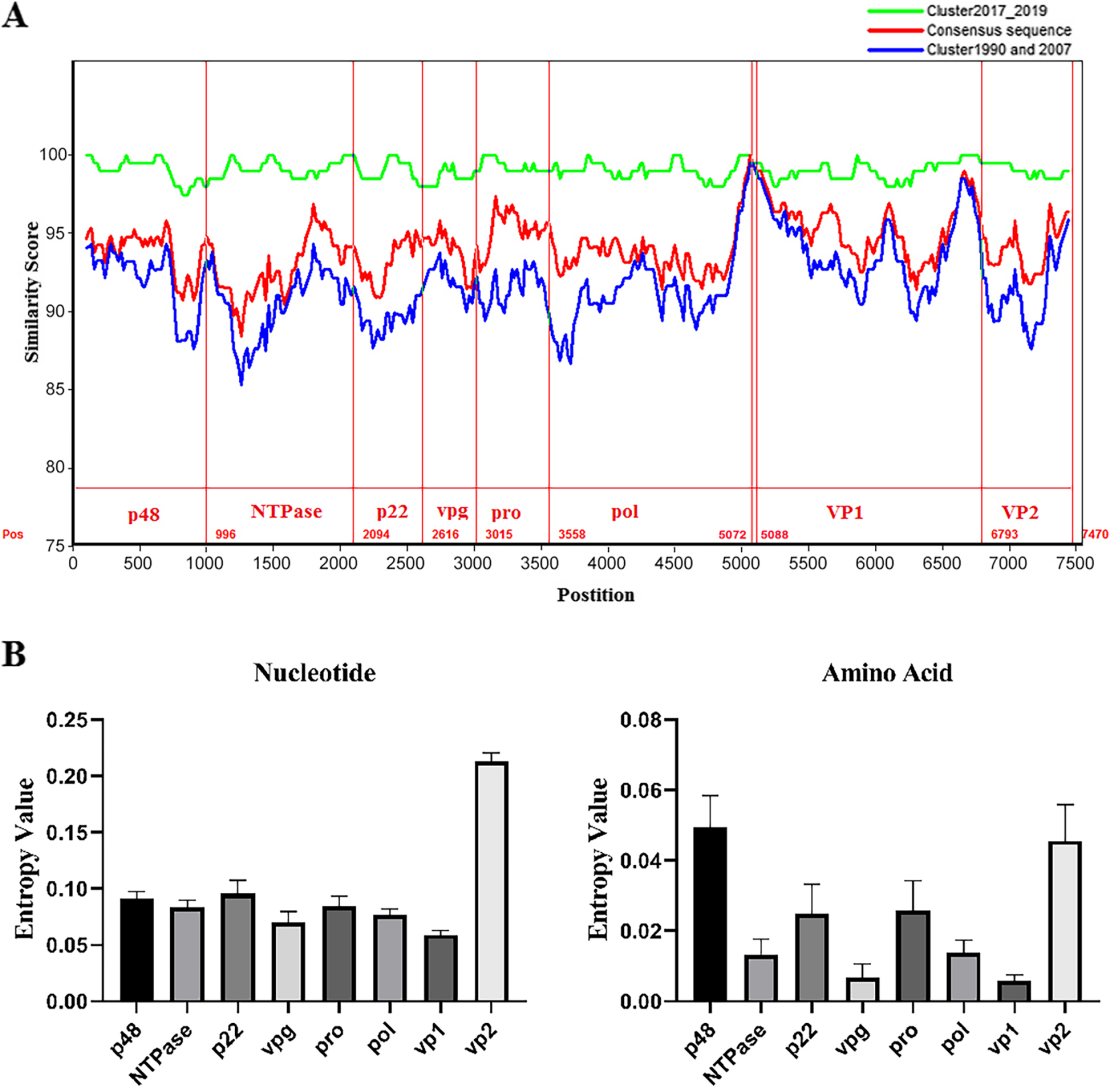
Comparative sequence analysis of GIX.1[GII.P15] strains. **A** Similarity plot analysis of whole-genome nucleotide sequence of KMN1 strain compared with the GIX.1[GII.P15] strains from NCBI. **B** Genetic variability of encoding regions was calculated at nucleotide and amino acid level using Shannon entropy for GIX.1[GII.P15] norovirus. Bars represent the mean value calculated from individual residue values. Standard errors are shown for each bar

To compare the genetic diversity of KMN1 with other GIX.1[GII.P15] strains, the complete nucleotide and amino acid sequences of the KMN1 strain was compared with other completely sequenced GIX.1[GII.P15] strains available from NCBI. Among these strains, 3 were detected from China, 8 from the USA, 1 from Japan, and 14 from Saudi Arabia. Overall, percent nucleotide identities between KMN1 strain and other strains displayed 94.63 ± 3.04 similarity in the full-length sequence and 94.15% ± 3.54% similarity in the VP1 region (Supplementary Table S1). Within group, further nucleotide similarity analysis showed that KMN1 is most closely related to the 2017–2019 subcluster, while demonstrating more divergence with the 1990–2007 subcluster and the consensus sequence (Fig. 4A). The consensus sequence of the GIX.1[GII.P15] genotype was constructed from the most frequent nucleotides or amino acid residues at each site of 26 other completed sequenced GIX.1[GII.P15] genotype strains available from NCBI. Alignment analysis of nucleotide sequences of the complete genome of KMN1 strain with the consensus sequence of the GIX.1[GII.P15] genotype showed 96 nucleotide substitutions across the full-length genome. Among all substitutions, 25 (25/96, 26.04%) were found in the VP1 region, of which, 6 substitutions were included in the P1 domain (A1284G, A1287G, T1395C, A1452G, T1485C, A1545G), 9 in the P2 domain (C840T, T864C, C903T, C904T, A921G, C972T, C1092T, A1167G, T1191C) and 7 in the S domain (G174A, A240G, A255G, T366C, A417T, C465T, G651A). The nucleotide differences between KMN1 and the consensus sequence are shown in detail in Table 1.

**Table 1:** Comparison of KMN1 nucleotide substitutions with the consensus sequences of GIX.1[GII.P15] strains

Gene	**p48**													
Position	**170**	**251**	285	420	504	657	723	735	819	831	834	843	855	900
Consensus	**C**	**A**	G	C	A	C	C	C	G	G	G	C	A	A
KMN1	**T**	**G**	A	T	G	T	T	T	A	T	A	A	G	G
Gene		**NTPase**												**p22**
Position	906	63	69	306	345	489	615	660	663	744	924	930	957	144
Consensus	C	A	T	C	C	T	C	A	C	G	C	C	G	A
KMN1	T	G	C	T	T	G	T	G	T	A	T	T	A	G
Gene				**VPg**								**Pro**		
Position	150	432	441	18	24	57	108	162	**277**	330	342	9	24	150
Consensus	C	A	G	C	G	C	G	T	**G**	C	T	G	C	T
KMN1	T	G	T	T	A	T	A	C	**A**	T	C	A	T	C
Gene					**Pol**									
Position	252	306	483	541	21	174	177	504	564	**634**	639	807	810	846
Consensus	T	C	A	T	A	A	C	A	C	**A**	G	G	A	G
KMN1	C	T	G	C	G	G	T	G	T	**G**	A	A	G	A
Gene								**VP1**						
Position	1059	1095	1098	1284	1314	1344	1395	102	174	240	255	366	417	465
Consensus	G	T	A	G	C	G	G	G	G	A	A	T	A	C
KMN1	A	C	G	A	T	A	A	A	A	G	G	C	T	T
Gene														
Position	651	676	687	840	864	903	**904**	921	972	1092	1167	1191	1284	1287
Consensus	G	T	T	C	T	C	**C**	A	C	C	A	T	A	A
KMN1	A	C	C	T	C	T	**T**	G	T	T	G	C	G	G
Gene					**VP2**									
Position	1395	1452	1485	1545	138	315	330	**491**	558	621	663	687		
Consensus	T	A	T	A	C	T	A	**C**	C	C	T	A		
KMN1	C	G	C	G	T	C	G	**T**	T	T	C	G		

Positions in bold represent nucleotide changes that resulted in changes in the amino acid sequence

To further evaluate the genetic variability, Shannon entropy for each encoding region of GIX.1[GII.P15] strains was calculated at both the nucleotide and amino acid level. The results revealed that within GIX.1[GII.P15] genotype, non-structural and VP2 proteins presented higher diversity than VP1 protein at both nucleotide and amino acid level (Fig. 4B and C). Further alignment analysis of amino acid sequences indicated that most nucleotide substitutions (90/96, 93.75%) were synonymous mutations, and 6 substitutions were non-synonymous mutations that cause amino acid substitutions. Among the nonsynonymous mutations, 2 mutations were in p48 (T57I and H84R); 1 was in VPg (V93I); 1 was in Pol (M212V), 1 was in VP1 (P302S), and 1 were in VP2 region (T164I) (Table 2). Notably, two amino acid sites (M212V in Pol and T164I in VP2) were found to be specific to the KMN1 strain, which have not been reported in other GIX.1[GII.P15] strains.

**Table 2:** Differences in the deduced amino acid sequence alignment of the KMN1 strain

Amino Acid site	P48		VPg	Pol	VP1	VP2
	57	84	93	212	302	164
Consensus Sequence	T	H	V	M	P	T
**MT707683.1|China|KMN1**	**I**	**R**	**I**	**V**	**S**	**I**
MT703831.1|China|07-Jun-2019	T	H	V	M	P	T
MN473468.1|China|14-May-2018	V	R	I	M	S	T
MN462922.1|China|13-Mar-2018	V	R	I	M	S	T
MN227774.1|USA|07-Jul-2018	I	H	V	M	P	T
MN227775.1|USA|18-May-2018	T	H	V	M	P	T
MN227777.1|USA|09-May-2018	I	H	V	M	P	T
MN227776.1|USA|19-Jan-2018	I	R	V	M	S	T
MN227771.1|USA|27-Dec-2017	I	H	V	M	P	T
MN227773.1|USA|15-Dec-2017	I	H	V	M	P	T
MN227772.1|USA|14-Dec-2017	I	H	V	M	P	T
MN227770.1|USA|14-Dec-2017	I	R	V	M	S	T
NC_044045.1|Japan|2007	T	H	V	M	P	T
MW261797.1| DS379|1990	T	H	V	M	P	T
MW261794.1| DS384|1990	T	H	V	M	P	T
MW261793.1| DS385|1990	T	H	V	M	P	T
MW261800.1| DS335|1990	T	H	V	M	P	T
MW261796.1| DS381|1990	T	H	V	M	P	T
MW261791.1| DS401|1990	T	H	V	M	P	T
MW261789.1| DS413|1990	T	H	V	M	P	T
MW261788.1| DS414|1990	T	H	V	M	P	T
MW261787.1| DS428|1990	T	H	V	M	P	T
MW261792.1| DS398|1990	T	H	V	M	P	T
MW261790.1| DS402|1990	T	H	V	M	P	T
MW261795.1| DS383|1990	T	H	V	M	P	T
MW261799.1| DS357|1990	I	H	V	M	P	T
MW261798.1| DS359|1990	I	H	V	M	P	T

### Prediction of conformational epitopes on the VP1 structure of GIX.1[GII.P15] strain

**Fig. 5:**
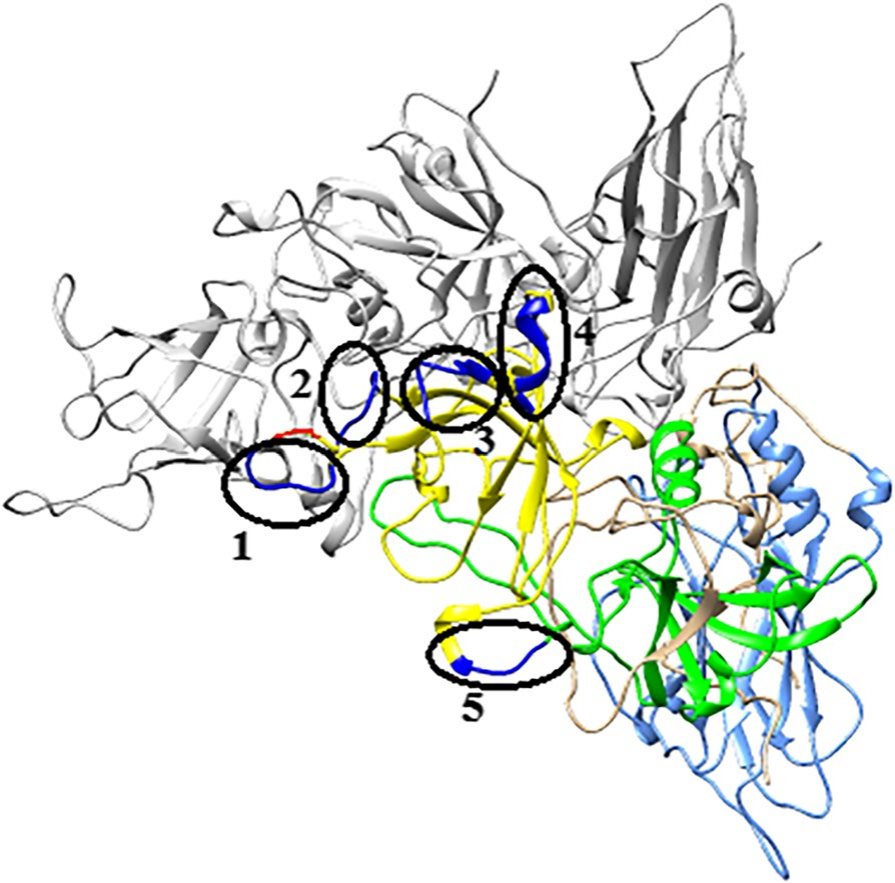
The three-dimensional VP1 dimer structures (cartoon models) of the GIX.1[GII.P15] strain are shown. Predicted epitopes of the KMN1 strain are indicated in dark blue, and their regions are circled with black (Region1:291,293,295-297aa; Region 2: 303–308 aa; Region 3: 349–357 aa; Region 4:388-393aa; Region 5:405-409aa); red: P302S substitution; light blue: S domain; Green: P1 domain; Yellow: P2 domain

Since the main neutralizing antibody epitopes of norovirus are located on the VP1 protein, and the antigenicity of the novel strain may be changed due to the mutations occurred on the VP1 protein, it is important to estimate the conformation epitopes and amino acid substitutions on the VP1 protein of GIX.1[GII.P15] strain. Here, we identified five regions as conformation epitopes by using computational methods [26, 27], four of which were located on the P2 domain and one was on the P1 domain (Fig. 5). Of note, the amino acid substitution in VP1 (P302S) was estimated around one of the conformational epitopes.

## Discussion

Norovirus is one of the most common causes of acute gastroenteritis in people of all ages worldwide. Most previous studies of norovirus have focused on epidemic strains such as GII.4 and GII.17 [28–31]. However, other genotypes can also cause outbreaks [32–34]. Thus, it is also important to study the genomic characteristics and monitor the mutations of minor strains. Here, we isolated a GIX.1[GII.P15] strain using Raji cells from a female patient in Kunming, China, and performed a comparative genomic analysis with other GIX sequences available in the public domain.

Phylogenetic analysis based on the VP1 and RdRp region indicated that KMN1 belonged to GIX.1[GII.P15] genotype clustering closely with two GIX.1[GII.P15] strains, both of which were collected in China in early 2018. Globally, GIX.1[GII.P15] is a rare genotype with a low detection rate [35–38], but this genotype has been reported to cause large outbreaks in US troops deployed to Saudi Arabia in 1990. Since then, only two subclusters were identified on the phylogenetic tree, suggesting a relatively static nature in the evolution of GIX.1[GII.P15] strains. In addition, the ORF1 gene (GII.P15) is most cloesley related with GII.P6 polymerase type, which could suggest that the GIX.1[GII.P15] strains might have diverged from this genotype.

Unlike GII.4 noroviruses, GIX.1[GII.P15] strains presented the lowest variation as compared with non-structural and VP2 regions, suggesting a low genetic robustness to adapt changes on their VP1 protein of this genotype. Further analysis of the full nucleotide sequences of KMN1 and the consensus sequence of GIX.1[GII.P15] strain revealed a total of 96 nucleotide substitutions in the full-length genome sequence, and only 6 of these substitutions resulted in amino acid sequence changes. Meanwhile, these sites were found as the differences within the two subclusters, suggesting that the 2017–2019 GIX.1[GII.P15] subcluster presented more diversity after 10 years of circulation in the human population and these sites maybe still evolve.

Of note, one amino acid substitution (P302S) was found in the P2 domain of VP1 protein, which is the highly variable region and the most exposed region of the structure [39]. Previous studies have shown that the variations in VP1 protein is of great significance to the evolution and epidemic of norovirus [34, 40]. Therefore, it’s likely that these alterations in the VP1 protein of this GIX.1[GII.P15] strain, together with the variations in non-structural and VP2 proteins, might endow new biological properties that enable this new strain escape human immune system or offer evolutionary advantages for infection or rapid spread via changing receptor binding sites or antibody recognition sites [41]. However, this S302 amino acid site was not located within the amino acid sequences of the HBGA-binding sites. The GIX.1[GII.P15] genotype has seven conserved residues that form the major components of the HBGA-binding sites [42]. The alignment of HBGA-binding pocket amino acid sequences in KMN1 strain and other GIX.1[GII.P15] strains showed very high identity and none of these residues were mutated in the GIX.1[GII.P15] strains in this study, indicating the HBGA-binding pocket is conserved in GIX.1[GII.P15] strains. In addition, two amino acid substitutions (M212V in RdRp and T163I in VP2) were found to be specific to the KMN1 strain. Since the number of full-length genome sequences of GIX.1[GII.P15] strains are still limited, further experiments are required to explore the effects of those mutations on HuNoV evolution and biology.

Finally, the predicted conformational epitopes were analyzed using computational methods and then mapped to the VP1 protein structure of the GIX.1[GII.P15] strain. Previous studies on other genotypes indicate that most epitopes have been predicted within the P2 domain and amino acid substitutions arising in the epitopes might change the antigenicity of these genotypes [9, 43]. Likewise, our results showed that four of the five predicted epitopes were located on the P2 domain, while the remaining one epitope was located on the P1 domain. Note that the P302S mutation in the P2 domain was predicted around one of the epitopes, which may confer new antigenic characteristics to the GIX.1[GII.P15] strain. And above all, these results also indicated that GIX.1[GII.P15] strain has evolved with limited alteration of their antigenicity.

## Conclusions

In summary, we report a full-genome sequence analysis of a rare norovirus GIX.1[GII.P15] strain from China. The genome information obtained from the KMN1 strain is important to better understand the genetic diversity, epidemiology and evolution of GIX.1[GII.P15] strains and will provide critical information for prevention and control GIX.1[GII.P15]-related outbreaks.

## Supplementary Information

**Additional file 1: Fig. S1.** The Phylogenetic tree based on full-genome sequences with different genotype reference strains. The GIX.1[GII.P15] strain identified in this study is indicated with a solid black circle. Bootstrap values greater than 75% are shown on the corresponding branches.

**Additional file 2: Table S1.** Percent Nucleotide identity (PNI) of full-length sequence and ORF1, ORF2 and ORF3 between the KMN1 strain and other GIX.1[GII.P15] strains available in GenBank.

## Data Availability

The full genome sequence of KMN1 strain described in the current study can be freely and openly accessed on NCBI database (https://www.ncbi.nlm.nih.gov/nucleotide/) under the accession number MT707683.1 and all data generated or analyzed during this study are included in this article.

## Abbreviations

HuNoV: Human norovirus
RdRp: RNA-dependent RNA polymerase
qRT-PCR: Real-time reverse transcription-PCR
ORFs: Open reading frame
HBGA: Histo-blood group antigens
TEM: Transmission electron microscope
