# Supplementary material for: Identification and genetic characterization of a minor norovirus genotype, GIX.1[GII.P15], from China
Source: BMC Genom Data. 2022 Jul 6;23:50. doi: 10.1186/s12863-022-01066-6 (PMC9261040; doi:10.1186/s12863-022-01066-6)

**Fig. S1, The Phylogenetic tree based on full-genome sequences with different genotype reference strains.** The GIX.1[GII.P15] strain identified in this study is indicated with a solid black circle. Bootstrap values greater than 75 % are shown on the corresponding branches.


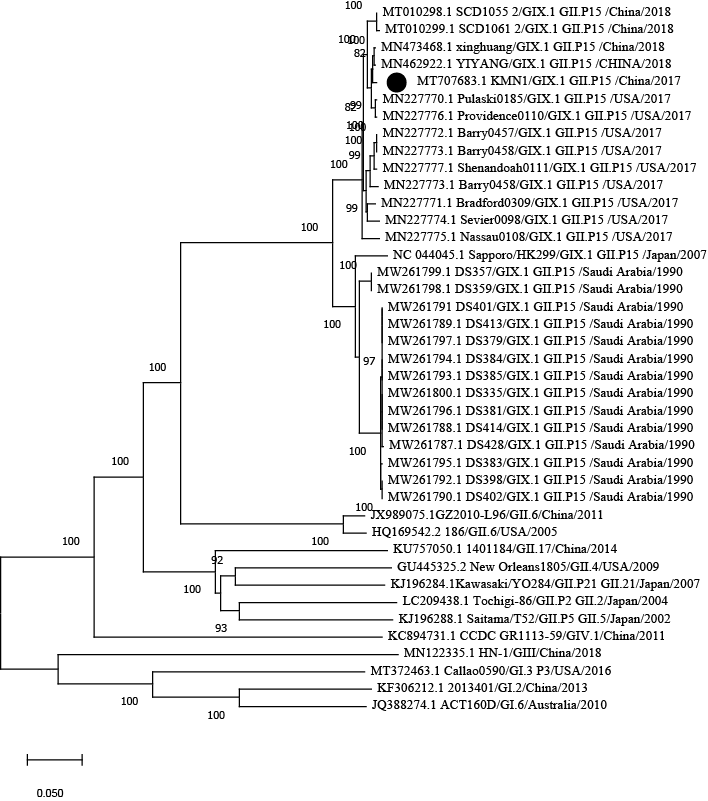

Supplement: Supplementary file 1 — Additional file 1: Fig. S1. The Phylogenetic tree based on full-genome sequences with different genotype reference strains. The GIX.1[GII.P15] strain identified in this study is indicated with a solid black circle. Bootstrap values greater than 75% are shown on the corresponding branches. [file 12863_2022_1066_MOESM1_ESM.docx]
