# Supplementary material for: Identification and genetic characterization of a minor norovirus genotype, GIX.1[GII.P15], from China
Source: BMC Genom Data. 2022 Jul 6;23:50. doi: 10.1186/s12863-022-01066-6 (PMC9261040; doi:10.1186/s12863-022-01066-6)
Supplement: Supplementary file 2 — Additional file 2: Table S1. Percent Nucleotide identity (PNI) of full-length sequence and ORF1, ORF2 and ORF3 between the KMN1 strain and other GIX.1[GII.P15] strains available in GenBank. [file 12863_2022_1066_MOESM2_ESM.docx]

Table S1

Percent Nucleotide identity (PNI) of full-length sequence and ORF1, ORF2 and ORF3 between the KMN1 strain and other GIX.1[GII.P15] strains available in GenBank.

| Strain  (GenebankID\|Country\|Collection_Date) | PNI of full-length sequence  (%) | PNI of ORF1 sequence (%) | PNI of ORF1 sequence (%) | PNI of ORF1 sequence (%) |
| --- | --- | --- | --- | --- |
| MT703831.1\|China\|07-Jun-2019 | 97.4 | 97.4 | 97.4 | 97.2 |
| MN473468.1\|China\|14-May-2018 | 99.2 | 99.3 | 99.2 | 98.4 |
| MN462922.1\|China\|13-Mar-2018 | 99.2 | 99.3 | 99.4 | 97.9 |
| MN227774.1\|USA\|07-Jul-2018 | 96.7 | 97.5 | 97.4 | 91.3 |
| MN227775.1\|USA\|18-May-2018 | 97.2 | 97.3 | 97 | 96.9 |
| MN227777.1\|USA\|09-May-2018 | 97.7 | 97.8 | 97.6 | 97.3 |
| MN227776.1\|USA\|19-Jan-2018 | 98.8 | 99 | 99.1 | 97.4 |
| MN227771.1\|USA\|27-Dec-2017 | 97.9 | 97.9 | 97.8 | 98.1 |
| MN227773.1\|USA\|15-Dec-2017 | 97.7 | 97.6 | 97.6 | 98 |
| MN227772.1\|USA\|14-Dec-2017 | 97.7 | 97.6 | 97.6 | 98.2 |
| MN227770.1\|USA\|14-Dec-2017 | 99 | 99 | 99.2 | 98.7 |
| NC_044045.1\|Japan\|2007 | 91.4 | 90.5 | 92.8 | 93.6 |
| MW261797.1\| DS379\|1990 | 92.2 | 91.3 | 94.3 | 93.5 |
| MW261794.1\| DS384\|1990 | 92.2 | 91.3 | 94.3 | 93.5 |
| MW261793.1\| DS385\|1990 | 92.2 | 91.3 | 94.3 | 93.5 |
| MW261800.1\| DS335\|1990 | 92.2 | 91.3 | 94.3 | 93.5 |
| MW261796.1\| DS381\|1990 | 92.2 | 91.3 | 94.3 | 93.5 |
| MW261791.1\| DS401\|1990 | 92.2 | 91.3 | 94.3 | 93.3 |
| MW261789.1\| DS413\|1990 | 92.2 | 91.3 | 94.3 | 93.3 |
| MW261788.1\| DS414\|1990 | 92.2 | 91.3 | 94.3 | 93.3 |
| MW261787.1\| DS428\|1990 | 92.2 | 91.2 | 94.3 | 93.3 |
| MW261792.1\| DS398\|1990 | 92.1 | 91.2 | 94.1 | 93.1 |
| MW261790.1\| DS402\|1990 | 92.1 | 91.2 | 94.1 | 92.8 |
| MW261795.1\| DS383\|1990 | 92.1 | 91.1 | 94.3 | 93.1 |
| MW261799.1\| DS357\|1990 | 92.2 | 91.3 | 94.3 | 93.2 |
| MW261798.1\| DS359\|1990 | 92.2 | 91.3 | 94.3 | 92.9 |
